# A novel super-resolution contrast-enhanced ultrasound approach for evaluating inflammatory activity in Crohn’s disease

**DOI:** 10.1186/s13244-026-02309-1

**Published:** 2026-05-20

**Authors:** Ying Wang, Wensong Ge, Yi Yu, Li Wei, Wenjun Ding, Rui Cheng, Yunlin Huang, Yi Dong, Peng Du

**Affiliations:** 1 Department of Ultrasound, Xinhua Hospital Affiliated to Shanghai Jiaotong University School of Medicine, Shanghai, China; 2 Department of Gastroenterology, Xinhua Hospital Affiliated to Shanghai Jiaotong University School of Medicine, Shanghai, China; 3 Department of Anorectal Surgery, Xinhua Hospital Affiliated to Shanghai Jiaotong University School of Medicine, Shanghai, China

**Keywords:** Super-resolution contrast-enhanced ultrasound (SR-CEUS), Inflammatory bowel disease, Inflammation activity, Quantitative evaluation

## Abstract

**Objectives:**

To investigate the role of super-resolution contrast-enhanced ultrasound (SR-CEUS) in evaluating inflammatory activity in Crohn’s disease (CD).

**Materials and methods:**

In this prospective study, we consecutively enrolled CD patients confirmed by clinical and ileocolonoscopic findings. All patients underwent B-mode ultrasound (BMUS), color Doppler flow imaging (CDFI), CEUS, and SR-CEUS within 1 week of ileocolonoscopy. SR-CEUS quantitative parameters were recorded, with simple endoscopic score for Crohn’s disease (SES-CD) as the reference standard. Diagnostic performance was evaluated using receiver operating characteristic (ROC) curve analysis.

**Results:**

52 consecutive CD patients were categorized into active (SES-CD ≥ 3, *n* = 30) and inactive (SES-CD < 3, *n* = 22) groups. SR-CEUS clearly visualized the intramural microvascular architecture of the bowel wall. SR-CEUS yielded an AUC of 0.903 with 86.4% sensitivity (95% CI: 66.7–95.3%), and 86.7% specificity (95% CI: 70.3–94.5%) for assessing inflammatory activity, significantly outperforming both CDFI (*p* = 0.014) and CEUS (*p* = 0.045), while showing no statistically significant difference in comparison with BMUS (*p* = 0.988). Furthermore, the combination of BMUS and SR-CEUS achieved an AUC of 0.967 for diagnosing active CD, with 100% sensitivity (95% CI: 85.1–100%) and 86.7% specificity (95% CI: 70.3–94.7%), which was significantly superior to BMUS alone (*p* = 0.038).

**Conclusions:**

SR-CEUS provides quantitative microvascular perfusion maps that display vascular density, flow velocity, and direction, offering a non-invasive tool for evaluating inflammatory activity in CD.

**Critical relevance statement:**

This study demonstrates that super-resolution contrast-enhanced ultrasound (SR-CEUS) provides a novel, non-invasive approach for quantitative evaluation of inflammatory activity in Crohn’s disease (CD), which serves as a valuable supplement or alternative to endoscopy in routine monitoring.

**Key Points:**

- An unmet need remains for accurate, non-invasive tools to assess CD activity.
- SR-CEUS outperforms conventional CDFI and CEUS in distinguishing active from inactive CD.
- Combining SR-CEUS with standard BMUS yields excellent diagnostic accuracy, establishing this combined approach as a promising non-invasive alternative for monitoring inflammatory activity in CD patients.

**Graphical Abstract:**

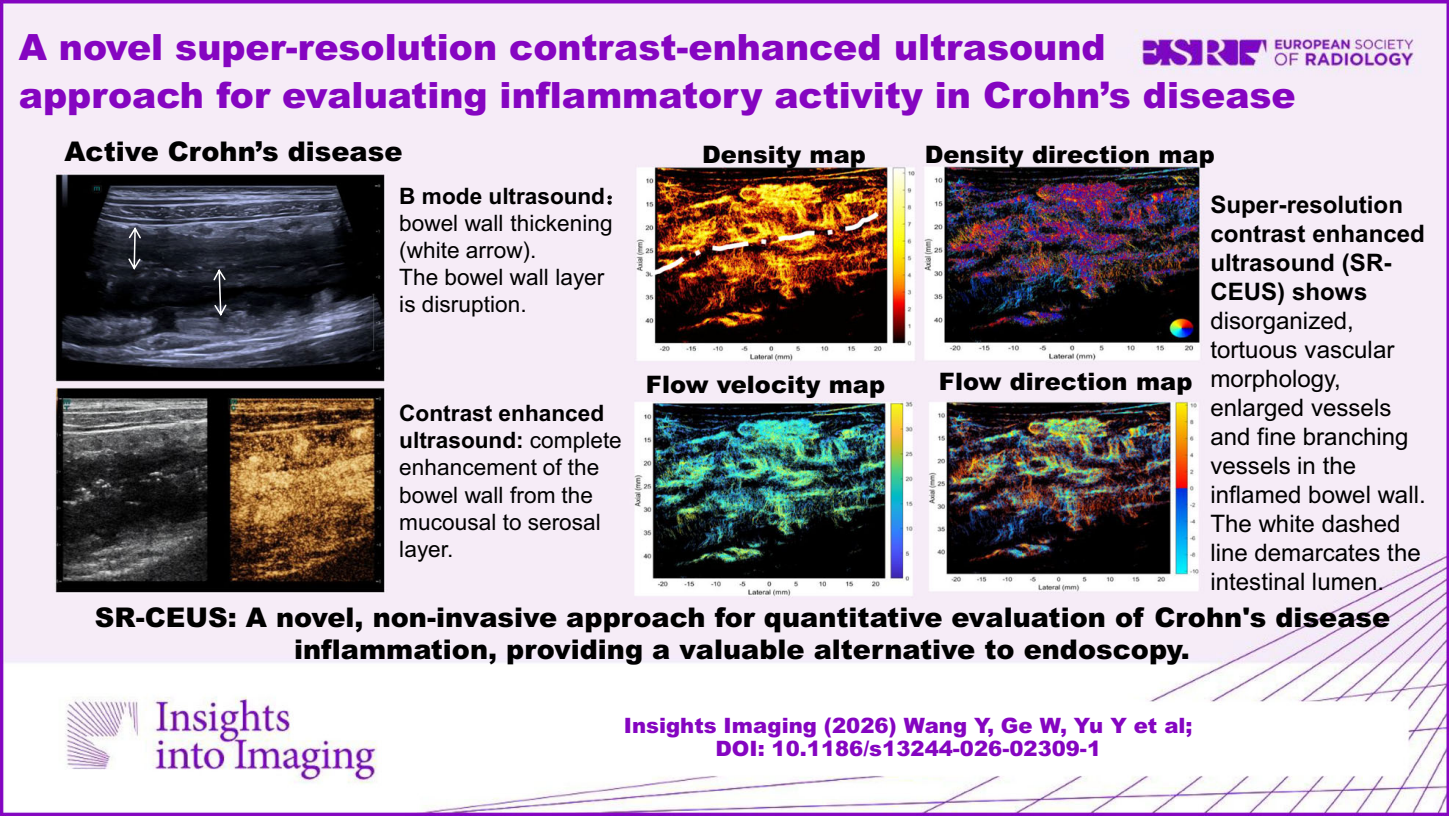

## Introduction

According to the 2025 European Crohn’s and Colitis Organization and the European Society of Gastrointestinal and Abdominal Radiology (ECCO-ESGAR) guidelines, Crohn’s disease (CD) is categorized as active or inactive based on clinical, endoscopic, and biochemical evidence [1]. Active CD may lead to severe complications such as fistulas, abscesses, and ileus [2]. While ileocolonoscopy with the simple endoscopic score for CD (SES-CD) is the reference standard, it is invasive and limited in evaluating transmural and extraluminal disease [3, 4]. The 2025 ECCO-ESGAR guidelines recommend cross-sectional imaging techniques such as computed tomography enterography (CTE) and magnetic resonance enterography (MRE) for comprehensive assessment [1]. Reported diagnostic performance indicates that CTE has a pooled sensitivity of 93% and specificity of 67% for active CD [5], whereas MRE has a mean sensitivity of 75% and specificity of 91% [6]. However, CTE involves ionizing radiation exposure, and MRE is costly with long acquisition times [7].

The 2025 ECCO-ESGAR guidelines explicitly promote intestinal ultrasound over CTE for CD assessment, given its non-invasiveness, lack of ionizing radiation, and wide availability [8]. Conventional B-mode ultrasound (BMUS) evaluates bowel wall thickness (BWT), morphological changes, and intra-abdominal complications [9], while color Doppler flow imaging (CDFI) semi-quantitatively assesses intramural blood flow [3, 10]. However, BWT thickening is non-specific and can result from various conditions, including neoplasms, ischemia, and other systemic diseases [11]. Moreover, CDFI is operator-dependent with limited sensitivity for low-velocity or small-vessel blood flow [12]. Furthermore, standardized intestinal ultrasound activity scores have been developed to improve diagnostic objectivity [13]. Although small intestinal contrast ultrasonography (SICUS) improves luminal distension and lesion detection using oral contrast and shows good correlation with MRE, it is more time-consuming and logistically complex [14, 15]. Contrast-enhanced ultrasound (CEUS) enables real-time microvascular perfusion imaging and quantitative analysis via time-intensity curves [16], but its spatial resolution is still limited by acoustic diffraction limits [17].

Super-resolution CEUS (SR-CEUS) has recently been developed to overcome this limitation by localizing and tracking individual microbubbles, enabling high-resolution microvascular imaging and quantitative hemodynamic analysis [18–20]. Although SR-CEUS has shown potential in other organs and diseases, its value for assessing intestinal inflammation in CD remains largely unexplored [18, 21]. Therefore, this study aimed to investigate the additional diagnostic value of SR-CEUS for the quantitative evaluation of inflammatory activity in CD patients.

## Materials and methods

This prospective study was approved by the hospital’s ethics committee (ID No. XHEC-C-2023-038). Written informed consent was obtained from all patients. All procedures complied with the Declaration of Helsinki.

### Patients

**Fig. 1:**
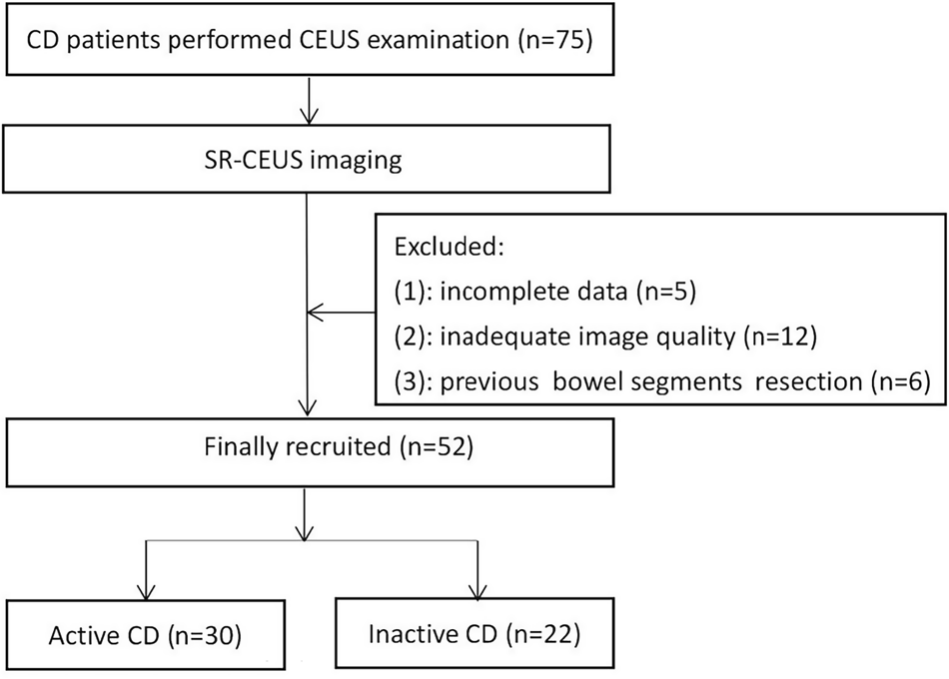
A flow diagram for the patients

Consecutive CD patients diagnosed based on clinical symptoms, laboratory and ileocolonoscopy were enrolled prospectively, with inclusion criteria: (1) aged 18–85 years; (2) having undergone BMUS, CDFI, CEUS, SR-CEUS, and ileocolonoscopy examinations; (3) CD activity assessed by SES-CD criteria; (4) the bowel wall could be clearly visualized during ultrasound. Exclusion criteria included: (1) incomplete clinical or imaging data; (2) inadequate image quality, including suboptimal CEUS images caused by intestinal peristalsis or gastrointestinal tract involvement; (3) a previous history of intestinal segment surgical resection. A flow diagram for the patients is presented in Fig. 1.

### Ultrasound examination technique

All ultrasound examinations were performed by a sonographer with over 10 years of intestinal ultrasound experience, using a Resona A20 ultrasound system (Mindray Medical Systems, China; SC7-1U, L18-5WU, SL10-3U probes) with SR-CEUS capability, all performed within 1 week of ileocolonoscopy.

#### Intestinal ultrasound examination

For BMUS, patients fasted for 8 h; the colon and small intestine were scanned systematically with the SC7-1U convex probe (1.2–6.0 MHz bandwidth) [10], and the thickest segment was examined with the L18-5WU linear probe (3.8–18.0 MHz) to assess BWT (with values > 3 mm defined as thickening), bowel wall stratification (normal/disrupted), mesenteric lymph nodes (MLN, enlarged if short-axis > 5 mm) and mesenteric fat hypertrophy (MFH, hyperechoic tissue surrounding inflamed segments) [3].

#### Color Doppler flow imaging examination

CDFI was performed on the thickest bowel wall segment with the L18-5WU probe, and blood flow signals were graded by the Limberg score [22]: Limberg 0, normal intestinal wall; Limberg I, no blood flow signals within the thickened bowel wall; Limberg II, dot- or short-linear blood flow signals detected in the thickened bowel wall; Limberg III, longer blood flow signals detected in the thickened bowel wall; Limberg IV, longer blood flow signals connected to the mesentery detected in the thickened bowel wall. Imaging was performed using standardized parameters: velocity scale < 10 cm/s, dynamic range of 25–30 dB, pulse repetition frequency (PRF) of 0.7 kHz, and wall filter < 102 Hz.

#### Contrast-enhanced ultrasound examination

CEUS was performed on the longitudinal section of the thickest intestinal segment using an SL10-3U probe (2.5–11.0 MHz bandwidth; mechanical index 0.08, gain 45 dB, dynamic range 110 dB, frame rate 10 fps). A bolus of 2.0 mL of the ultrasound contrast agent SonoVue (Bracco SpA) was administered intravenously, followed by 5.0 mL of saline flush. Real-time CEUS data were continuously acquired for ≥ 2 min with enhancement patterns classified into four categories according to the established criteria [23]. All ultrasound cine loops were digitally recorded in Digital Imaging and Communications in Medicine (DICOM) format for offline analysis.

#### Super-resolution contrast-enhanced ultrasound (SR-CEUS) examination

SR-CEUS was performed simultaneously with CEUS. During a brief breath-hold by patients, a 6-s cine loop of CEUS data was acquired at a maximum frame rate of 500 frames per second for SR-CEUS processing. Following a 30-s image reconstruction period, the SR-CEUS algorithm generated four distinct blood flow visualization maps: vascular density, vascular directionality, flow velocity, and flow direction. The acquired SR-CEUS raw data were then exported and analyzed offline using dedicated software (Version 2, Mindray Medical Systems). Various SR-CEUS quantitative parameters were derived from a manually drawn region of interest (ROI) of 2 cm within the most vascularized section of the thickest inflamed bowel wall. The analyzed parameters included vessel density (VD), flow weighted density of the vessel (FWDV), fractal dimension (FD), velocity (mm/s), velocity variance (Vel Var), velocity entropy (Vel Entropy), direction variance (Dir Var), direction entropy (Dir Entropy), and perfusion index (PI) [24].

Two trained sonographers (Sonographers 2 and 3) independently evaluated SR-CEUS qualitative parameters (blinded to clinical/pathological data), with discrepancies resolved by a third senior sonographer (Sonographer 4). Sonographer 2 evaluated quantitative parameters, and Sonographer 3 repeated the analysis for inter-operator reliability, with both being blinded to each other’s results.

### Ileocolonoscopy evaluation

Ileocolonoscopy was performed by endoscopists with over 10 years of IBD experience, and CD mucosal activity was assessed using the SES-CD score (active CD defined as SES-CD ≥ 3) [25, 26]. Score discrepancies were adjudicated by a senior endoscopist with 20 years of experience, whose assessment was used for final analysis.

### Statistical analysis

All data were analyzed using SPSS 26.0 and GraphPad Prism 9 (GraphPad Software Inc.). Continuous variables with a normal distribution were compared using the independent two-sample *t*-test, while those with a non-normal distribution were compared using the Mann–Whitney U test. Categorical variables were analyzed using the chi-square test. Optimal cutoffs, sensitivity, and specificity were defined by the maximum Youden index on ROC curves. Binary logistic regression generated predicted probabilities. The area under the curve (AUC) analysis assessed the diagnostic performance of ultrasound methods in evaluating inflammatory activity in CD patients based on SES-CD scores, with a paired-sample design comparing the AUCs. Intraclass correlation coefficient (ICC) assessed inter-operator reliability, with *p* < 0.05 considered statistically significant.

## Results

### Patients’ characteristics

From March to November 2024, 52 CD patients (21 females, 31 males; mean age 39.5 years, range 18–67 years) were enrolled and stratified by SES-CD into active (≥ 3, *n* = 30) and inactive (< 3, *n* = 22) groups. Regarding disease location, ileocolonic involvement was the most common phenotype in both the active (60.0%, 18/30) and inactive (50.0%, 11/22) CD groups (Table 1).

**Table 1:** Base characteristics of Crohn’s disease patients

	Active CD group (*n* = 30)	Inactive CD group (*n* = 22)	*p*-value
Gender			0.496
Female	11 (36.7%)	10 (45.5%)	
Male	19 (63.3%)	12 (54.5%)	
Age (years)			0.469
Mean ± SD	38.2 ± 14.4	41.1 ± 15.4	
Range	18–64	18–67	
BMI (kg/m^2^)			0.804
Mean ± SD	21.8 ± 3.8	21.0 ± 3.5	
Range	12.6–31.1	14.7–30.1	
CRP (mg/L)			0.015*
Mean ± SD	19.1 ± 24.5	14.7 ± 26.3	
Range	1–90	1–89	
ESR (mm/h)			0.419
Mean ± SD	22.6 ± 19.8	22.8 ± 26.9	
Range	2–87	2–93	
Location			0.764
Terminal ileum	8 (26.7%)	7 (31.8%)	
Colon	4 (13.3%)	4 (18.2%)	
Ileocolon	18 (60.0%)	11 (50.0%)	

*SD* standard deviation, *BMI* body mass index, *CRP* C-reactive protein, *ESR* erythrocyte sedimentation rate, *Location* location of the inflamed bowel segment

* *p* < 0.05

### Intestinal B-mode ultrasound and contrast-enhanced ultrasound features

On BMUS scanning, BWT was significantly greater in the active CD group than in the inactive CD group (7.2 mm ± 1.8 vs 3.9 mm ± 0.9, *p* < 0.001) with an optimal cut-off value of 4.6 mm for distinguishing the two groups.

**Fig. 2:**
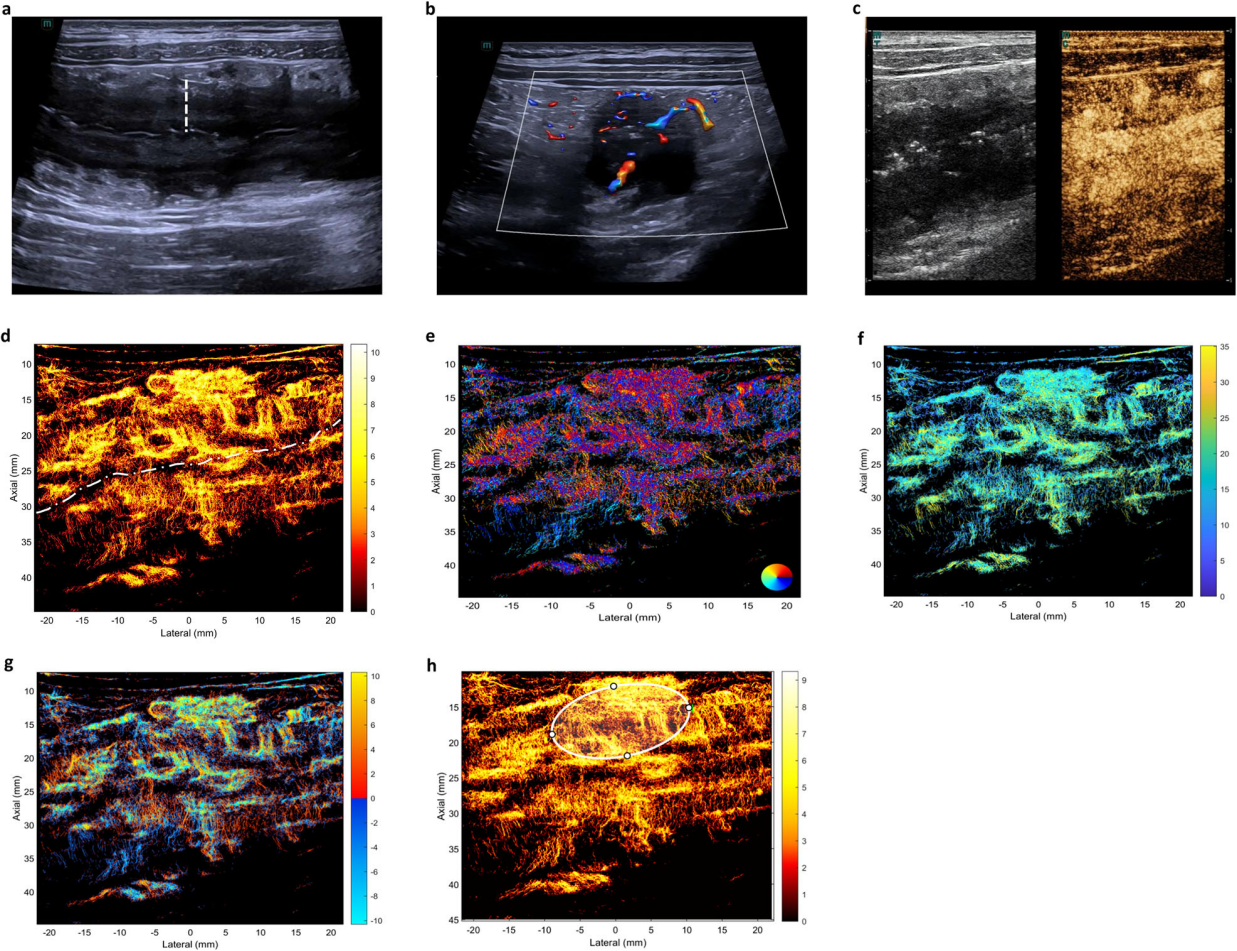
A 24-year-old female patient diagnosed with active Crohn’s disease (CD). The value of total simple endoscopy score for CD (SES-CD) is 13. On longitudinal scan, the bowel wall layers are disrupted. The bowel wall thickness of the descending colon is 11.0 mm (white dashed line, **a**); color Doppler signal shows longer linear blood flow signals extending to and connecting with the mesentery within the inflamed bowel wall (Limberg IV, **b**); contrast-enhanced ultrasound (CEUS) of the inflamed bowel wall displays complete enhancement of the bowel wall from the mucousal to serosal layer (pattern I, **c**); super-resolution CEUS (SR-CEUS) vascular density map of the inflamed bowel wall shows disorganized, tortuous vascular morphology, enlarged vessels and fine branching vessels the inflamed bowel wall. The white dashed line demarcates the intestinal lumen (**d**). The SR-CEUS vascular density direction map of the inflamed bowel wall is shown. Brighter colors indicate higher vascular density (**e**). The SR-CEUS flow velocity map of the inflamed bowel wall is shown. Variations in color on the scale bar represent different blood flow velocities (**f**). The SR-CEUS flow direction map of the inflamed bowel wall is shown. On the color scale, positive values are assigned to flow toward the probe, and negative values represent flow away from it (**g**). By SR-CEUS offline analyzing, quantitative parameters were automatically derived from a manually placed region of interest (ROI) over the inflamed bowel wall for objective assessment (**h**)

Regarding the bowel wall layer, disruption of the normal layered structure was observed in the majority of patients in the active CD group (66.7%, 20/30) (Fig. 2). In contrast, a clearly preserved layered structure was more commonly seen in the inactive CD group (72.7%, 16/22) (Fig. 2).

MFH, characterized by hyperechoic adipose tissue encircling the affected intestinal segment, was present in most patients in the active CD group (60.0%, 18/30) (Fig. 2, Table 2).

**Table 2:** Comparison of intestinal ultrasound features between the two groups

	Active CD group (*n* = 30)	Inactive CD group (*n* = 22)	*p*-value
BWT (mm)			< 0.001*
Mean ± SD	7.2 ± 1.8	3.9 ± 0.9	
Median (min, max)	7.5 (4.0, 11.4)	3.9 (2.1, 5.8)	
Layers of bowel wall			0.002*
Normal	10 (33.3%)	17 (72.3%)	
Disruption	20 (66.7%)	5 (22.7%)	
MLN			0.467
Normal	16 (53.3%)	14 (63.6%)	
Enlarged	14 (46.7%)	8 (36.4%)	
MFH			
Absent	12 (40.0%)	17 (77.3%)	0.007*
Present	18 (60.0%)	5 (22.7%)	
CDFI			0.038*
Limberg 0	0	2 (9.1%)	
Limberg I	0	1 (4.5%)	
Limberg II	9 (30.0%)	12 (54.5%)	
Limberg III	5 (16.7%)	3 (13.6%)	
Limberg IV	16 (53.3%)	4 (18.2%)	
CEUS enhancement			0.025*
Pattern I	16 (53.3%)	6 (27.3%)	
Pattern II	11 (36.7%)	7 (31.8%)	
Pattern III	3 (10.0%)	9 (40.9%)	
Pattern IV	0	0	

Color Doppler flow signals were classified according to the Limberg score: Limberg 0, normal intestinal wall; Limberg I, thickened bowel wall with no detectable blood flow signals; Limberg II, thickened bowel wall with dot- or short-like linear blood flow signals; Limberg III, thickened bowel wall with longer linear blood flow signals; Limberg IV, thickened bowel wall with longer linear blood flow signals extending to and connecting with the mesentery; CEUS enhancement patterns were classified four categories: pattern I, complete enhancement of the bowel wall from the mucosal to the serosal layer; pattern II, lack of enhancement in the outer border of the muscolaris propria; pattern III, enhancement confined exclusively to the submucosal layer; pattern IV, absence of enhancement throughout the entire intestine segment

*BWT* bowel wall thickness, *SD* standard deviation, *MLN* mesenteric lymph node, *MFH* mesenteric fat hypertrophy, *CDFI* color Doppler flow imaging

* *p* < 0.05

According to the Limberg score criteria, active CD predominantly showed Limberg IV (long flow signals connected to the mesentery, 53.3%, 16/30), while inactive CD primarily showed Limberg II (dot- or short-linear flow signals, 54.5%, 12/22, *p* = 0.038) (Table 2).

**Fig. 3:**
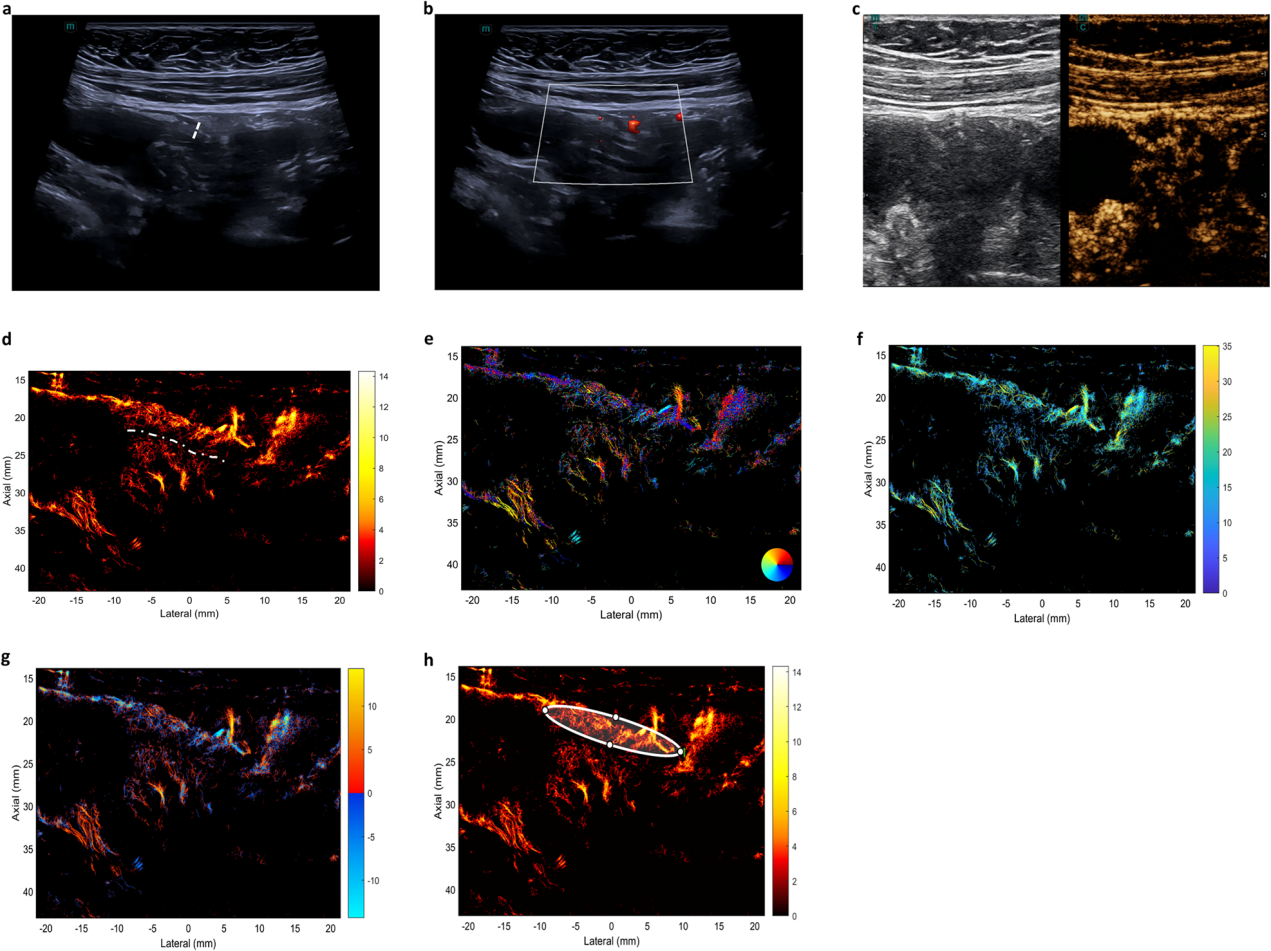
A 41-year-old female patient diagnosed with inactive Crohn’s disease (CD). The value of total simple endoscopy score for CD (SES-CD) is 1. On longitudinal scan, the bowel wall layers are clear. The bowel wall thickness of the ileum is 3.2 mm (white dashed line, **a**); color Doppler signal of the inflamed bowel wall displays dot-like blood flow signals (Limberg II, **b**); contrast-enhanced ultrasound (CEUS) of the thickest bowel wall displays enhancement of submucosa (pattern III, **c**); super-resolution CEUS (SR-CEUS) vascular density map of the inflamed bowel wall shows fine branching vessels in the inflamed bowel wall. The white dashed line demarcates the intestinal lumen (**d**). SR-CEUS vascular density direction map of the inflamed bowel wall is shown (**e**), SR-CEUS flow velocity map of the inflamed bowel wall is shown (**f**), SR-CEUS flow direction map of the inflamed bowel wall is shown (**g**). By SR-CEUS offline analyzing, quantitative parameters were automatically derived from a manually placed region of interest (ROI) over the inflamed bowel wall (**h**)

CEUS Pattern I (complete transmural enhancement) was the main pattern in active CD (53.3%, 16/30) (Fig. 2), while Pattern III (submucosa-only enhancement) prevailed in inactive CD (40.9%, 9/22, *p* = 0.025) (Fig. 3, Table 2).

### Super-resolution contrast-enhanced ultrasound (SR-CEUS) and SR-CEUS quantitative analysis

**Fig. 4:**
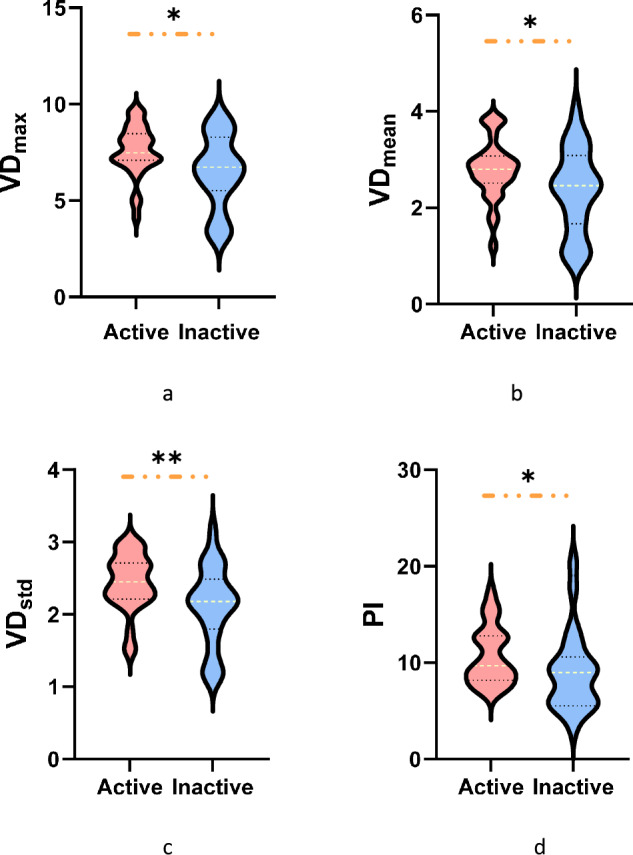
Comparison of super-resolution contrast-enhanced ultrasound (SR-CEUS) quantitative parameters between active and inactive CD groups. The red violin plots represent quantitative parameters of patients in active CD, while quantitative parameters of patients in inactive CD are expressed in the blue violin plots. The figures display significant differences between the two groups for the parameters, including maximum density value (VD_max_) (**a**), mean density value (VD_mean_) (**b**), standard deviation of density value (VD_std_) (**c**), and perfusion index (PI) (**d**), (* *p* < 0.05, ** *p* < 0.01)

SR-CEUS achieved high-resolution visualization of intestinal microvasculature and hemodynamics, with four switchable parametric maps (vascular density, vascular directionality, flow velocity, flow direction). Active CD exhibited disorganized, tortuous microvasculature with enlarged vessels and aberrant branching in inflamed bowel walls (Fig. 2). Among SR-CEUS quantitative parameters, VDmax, VDmean, VDstd, and PI were significantly higher in the active group than in the inactive group (*p* = 0.01, 0.022, 0.009, 0.046, respectively) (Fig. 4).

### Inter-operator reliability of the quantitative parameters

The inter-operator reliability of the quantitative parameters was excellent, with ICCs exceeding 0.900. The ICCs of the VD_max_, VD_mean_, VD_min_, VD_std_, FWDV, FD, V_max_, V_mean_, V_min_, Vel Var, Vel Entropy, Dir Var, Dir Entropy, and PI were 0.997 (95% CI: 0.994–0.998), 0.990 (95% CI: 0.982–0.994), 1.0 (95% CI: 0.999–1.0), 0.973 (95% CI: 0.954–0.984), 0.976 (95% CI: 0.958–0.986), 0.934 (95% CI: 0.884–0.962), 0.990 (95% CI: 0.983–0.994), 0.986 (95% CI: 0.975–0.992), 0.995 (95% CI: 0.991–0.997), 0.994 (95% CI: 0.990–0.997), 0.947 (95% CI: 0.910–0.967), 0.995 (95% CI: 0.992–0.997), 0.923 (95% CI: 0.866–0.956), 0.981 (95% CI: 0.968–0.989) respectively.

### Diagnostic efficiency of B-mode ultrasound (BMUS), color Doppler flow imaging (CDFI), contrast-enhanced ultrasound (CEUS), and super-resolution CEUS (SR-CEUS)

**Fig. 5:**
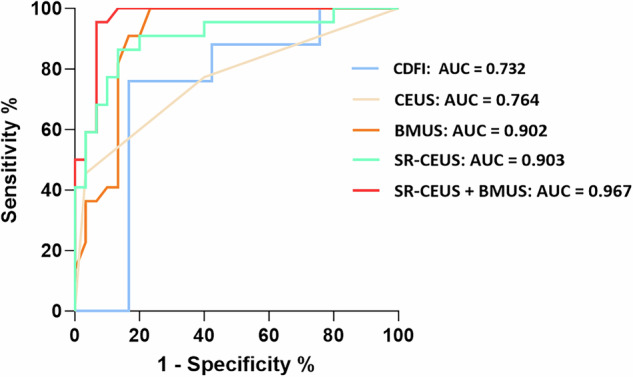
Receiver operating characteristic (ROC) curves analysis of the diagnostic performance of the different ultrasound imaging methods, including B-mode ultrasound (BMUS), color Doppler flow imaging (CDFI), contrast-enhanced ultrasound (CEUS) pattern, and the super-resolution CEUS (SR-CEUS) for evaluating the inflammatory activity of CD. The area under the ROC curve (AUC) of SR-CEUS in the diagnosis of active CD was 0.903, significantly higher than that of CDFI or CEUS (*p* < 0.05). The combined AUC of BMUS and SR-CEUS in the diagnosis of active CD was 0.967, significantly higher than that of BMUS alone (*p* = 0.038)

ROC analysis showed SR-CEUS had an AUC of 0.903 (95% CI: 0.814–0.992) for active CD, with a sensitivity of 86.4% (95% CI: 66.7–95.3%) and a specificity of 86.7% (95% CI: 70.3–94.5%), significantly outperforming CDFI (AUC = 0.732 [95% CI: 0.6–0.863], a sensitivity of 76.0% [95% CI: 56.6–88.5%], a specificity of 66.7% [95% CI: 49.6–80.3%], *p* = 0.014) and CEUS (AUC = 0.764 [95% CI: 0.627–0.902], a sensitivity of 45.5% [95% CI: 26.9–65.3%], a specificity of 96.7% [95% CI: 83.3–99.8%], *p* = 0.045). A direct comparison between the standalone diagnostic performance of BMUS (AUC 0.902) and SR-CEUS (AUC 0.903) showed no statistically significant difference (*p* = 0.988). The combination of BMUS and SR-CEUS yielded a higher AUC of 0.967 (95% CI: 0.923–1.0), a sensitivity of 100% (95% CI: 85.1–100%), a specificity of 86.7% (95% CI: 70.3–94.7%), which was significantly superior to BMUS alone (AUC 0.902, 95% CI: 0.815–0.989, a sensitivity of 90.9% [95% CI: 72.2–98.4%], a specificity of 83.3% [95% CI: 66.4–92.7%], *p* = 0.038) (Fig. 5, Table 3).

**Table 3:** Comparison of the diagnostic performance of different ultrasound methods in evaluating the inflammatory activity in CD patients

Imaging method	AUC (95% CI)	Sensitivity (95% CI) (%)	Specificity (95% CI) (%)	*Z* value	*p-*value
BMUS	0.902 (0.815–0.989)	90.9 (72.2–98.4)	83.3 (66.4–92.7)		
CDFI	0.732 (0.6–0.863)	76.0 (56.6–88.5)	66.7 (49.6–80.3)	2.452^a^	0.014*
CEUS	0.764 (0.627–0.902)	45.5 (26.9–65.3)	96.7 (83.3–99.8)	1.961^a^	0.045*
SR-CEUS	0.903 (0.814–0.992)	86.4 (66.7–95.3)	86.7 (70.3–94.5)	−0.015^b^	0.988
SR-CEUS + BMUS	0.967 (0.923–1.0)	100 (85.1–100)	86.7 (70.3–94.7)	−2.071^b^	0.038*

*BMUS* B-mode ultrasound, *CDFI* color Doppler flow imaging, *CEUS* contrast-enhanced ultrasound, *SR-CEUS* super-resolution CEUS, *AUC* area under the ROC curve

* *p* < 0.05

^a^ Comparing with the AUC value of SR-CEUS

^b^ Comparing with the AUC value of BMUS

## Discussion

The 2025 ECCO-ESGAR guidelines prioritize MRE and intestinal ultrasound as the cornerstones for a comprehensive CD assessment, underscoring the increasingly vital role of non-invasive imaging modalities in this context [1, 27]. Our findings confirm the clinical value of ultrasound. Most active CD patients in our cohort presented with characteristic BMUS features, including BWT thickening, disruption of bowel wall layers, and MFH. While BMUS alone demonstrated excellent diagnostic performance in our study (AUC 0.902), the potential for overlap with other pathologies suggests that integrating functional and quantitative parameters can further refine diagnostic accuracy [28].

Quantification of intestinal ultrasound findings is central to enhancing its objectivity and reproducibility, and standardized activity scores have advanced this field [29]. However, a key limitation of conventional intestinal ultrasound modalities is their indirect or semi-quantitative evaluation of microvascular changes that are a hallmark of active inflammation [30]. CDFI correlates with endoscopic activity, as demonstrated by Sævik et al (r = 0.64) [31]. The lower AUC of CDFI in our study (0.732) may be attributed to our cohort’s specific disease distribution, smaller sample size, and CDFI’s inherent limitations in detecting low-velocity flow in nascent microvessels [12].

CEUS uses microbubbles to better detect neovascularization and microperfusion within the bowel wall [32, 33], with active CD mostly showing Pattern I enhancement. CEUS provides real-time, direct visualization of microvascular perfusion dynamics in active CD [34]. Several studies have used CEUS parameters derived from the time-intensity curves, such as maximum peak intensity and relative peak enhancement, to assess CD inflammatory activity, yielding variable diagnostic efficiency (a sensitivity of 68–100% and specificity of 73–96% for active CD) [3]. However, its resolution is limited by diffraction, failing to visualize capillary-level microvessels clearly [17].

SR-CEUS represents a technological leap aimed at overcoming these barriers. To our knowledge, this is the first study applying SR-CEUS to CD inflammatory activity evaluation. By tracking individual microbubbles, SR-CEUS achieves capillary-scale visualization of intestinal microvasculature and hemodynamics [20, 24], revealing disorganized, tortuous vessels in active CD. The diagnostic performance analysis demonstrated that SR-CEUS achieved a significantly higher AUC compared to both CDFI and conventional CEUS. However, a direct comparison between SR-CEUS and BMUS alone revealed no statistically significant difference (*p* = 0.988), indicating that their standalone diagnostic accuracies for distinguishing active CD are equivalent. The superior performance of the combined BMUS and SR-CEUS model (AUC 0.967) underscores their complementary roles: BMUS provides essential morphological assessment, while SR-CEUS adds unique functional and microvascular characterization, together offering enhanced diagnostic value. This novel quantitative method can be incorporated into diagnostic workflows as a second-line ultrasound adjunct for CD, especially when endoscopy is contraindicated, or functional data is needed, delivering objective visual quantification of inflammatory activity.

This study has limitations: a single-center design, small sample size, and selection bias (only well-visualized bowel segments included), plus intestinal peristalsis impairing SR-CEUS image stability and reconstruction. Larger multicenter prospective studies are needed to validate these findings, standardize acquisition protocols, and optimize SR-CEUS parameter integration into clinical decision-making.

In summary, SR-CEUS generates quantitative microvascular parametric maps, addressing key limitations of conventional ultrasound and providing a powerful non-invasive tool for CD inflammatory activity evaluation, which promises to enhance clinical diagnostic confidence and monitoring precision.

## Data Availability

The datasets generated and analyzed during the current study are not publicly available due to patient confidentiality but are available from the corresponding author on reasonable request.

## Abbreviations

AUC: Area under the curve
BMUS: B-mode ultrasound
BWT: Bowel wall thickness
CD: Crohn’s disease
CDFI: Color Doppler flow imaging
CEUS: Contrast-enhanced ultrasound
CI: Confidence interval
Dir Var: Direction variance
FD: Fractal dimension
FWDV: Flow weighted density of the vessel
MFH: Mesenteric fat hypertrophy
PI: Perfusion index
ROC: Receiver operating characteristic
SD: Standard deviation
SES-CD: Simple endoscopic score of CD
SR-CEUS: Super-resolution CEUS
VD: Vessel density
Vel Var: Velocity variance
